# Silicon alleviates salinity stress in licorice (*Glycyrrhiza uralensis*) by regulating carbon and nitrogen metabolism

**DOI:** 10.1038/s41598-020-80739-7

**Published:** 2021-01-13

**Authors:** Jiajia Cui, Enhe Zhang, Xinhui Zhang, Qi Wang

**Affiliations:** 1 College of Agronomy, Gansu Agricultural University, Lanzhou, 730070 China; 2 College of Pharmacy, Key Laboratory of Hui Ethnic Medicine Modernization Ministry of Education, Ningxia Medical University, Yinchuan, 750004 China

**Keywords:** Ecology, Plant sciences

## Abstract

Salt stress is one of the key factors that limits the cultivation of *Glycyrrhiza uralensis* Fisch. (*G. uralensis*) in the northern part of China. In this study, three salt treatments (including 21, 42 and 63 ds/m NaCl/kg dry soil) and four Si (silicon) concentrations (including 0, 1.4, 2.8 and 4.2 ds/m SiO_2_/kg K_2_SiO_3_ in dry soil) were tested using *G. uralensis* as the plant material in a pot experiment with three replications. The results showed that the application of various concentrations of Si increased sucrose synthetase (SS), sucrose phosphate synthetase (SPS) and glutamine synthetase (GS), as well as nitrate reductase (NR) activities, and promoted carbon and nitrogen metabolism. Si application also increased the root dry weight of *G. uralensis*. Multilevel comparative analysis showed that the application of 2.8 ds/m SiO_2_ was the optimum rate for improved growth and yield of *G. uralensis* under different salt levels. This study provides important information that can form the basis for the cultivation of high-yielding and high-quality *G. uralensis* in saline soils.

## Introduction

Soil salinity is an abiotic stress that limits crop yield and productivity in an ecosystem. Soil salinity has been shown to affect approximately 800 million hectares of arable land worldwide^1^. It is currently receiving considerable attention in global agricultural research because of its significant threat to plant growth and development^2^. Other studies have shown that salt stress can alter a range of physiological and biochemical processes and inhibit enzyme activities of carbon (C) and nitrogen (N) metabolism^3^. This effect has been demonstrated among crops such as *Zea mays* L.^4^, tomato^5^, wheat^6^, chickpea^7^ and Ethiopian mustard^8^. Research indicates that salt stress affects the content of lipids, proteins and nucleic acids^9^ and changes the morphological, physiological, biochemical, and molecular characteristics of plants^10^. Studies however, indicate that salt stress significantly affects the fresh and dry weight of plants, which finally results in a decline in crop yield^11–14^. The constituents of medicinal plants can be changed in relation to their physiology and biochemistry prior to the effects of soil salinity which caused enormous yield losses^15^. Similarly, the negative effect of salt stress in medicinal plants has gradually been observed in *Matricaria chamomilla*^16^, *peppermint*^17^, *geranium*^18^, *Thymus vulgaris*^19^, *sage*^20^, and *Mentha pulegium*^21^.

Recently, the progresses and mechanisms of Si in alleviating various biotic and abiotic stresses in plants have been systematically reviewed by several researchers. Hence, an alternative strategy of Si supplementation to overcome the negative effects of salinity in plants can be considered as a valuable approach^22,23^. Although our understanding of the role of Si in abiotic stress resistance is still limited, there have been many important advances in its ability to alleviate salt and water stress, metal toxicity, and other adverse effects on plants^24–26^. Studies by Zhu et al.^27^ demonstrate that Si can enhance the survivability of crops under stress, such as grains, fruits, and vegetable crops. Zhang et al. confirmed the possible utility of Si in medicinal plants under salt stress and that the application of Si to medicinal plants can alleviate salinity stress^28^. Despite the ability of Si to decrease Na^+^ concentrations and improve antioxidant enzyme activity by harnessing the effect of salt stress on antioxidant responses, the effect of salt stress on the carbon (C) and nitrogen (N) metabolism of *G. uralensis* has not been studied^11^. To our knowledge, information about the effects of C and N metabolism responses to environmental conditions such as salt stress is very limited.

It is well known that C and N metabolism are very sensitive to environmental stress and closely related to nearly every biochemical pathway, as these elements are essential for plant growth and development^29^. As described by Zhang et al.^30^, NaCl stress has a strong influence on the metabolism of C and N, which is decreased the amount of these nutrients within the plant, the C and N metabolism are considered essential for the growth and development of plants. GS, NR, SS and SPS are the key enzymes catalyzing metabolites such as sugars, and proteins play important roles in C and N metabolic processes. Furthermore, salt stress significantly decreases the key enzyme activities, which are involved in the regulation of metabolism^31–33^. Hence, it is important to regulate key enzymes associated with plants to maintain an appropriate balance or ratio for C and N metabolism, which are important for plant growth and development under salt stress conditions^31^. However, little information is available regarding the influence of Si on C and N metabolism under salt stress.

*Glycyrrhiza uralensis* Fisch. is one of the most important medicinal plants in China because of its commercial value and patronage. Wild *Glycyrrhiza uralensis* Fisch. resources usually grow in arid, semiarid and saline regions and therefore have the ability to adapt to saline conditions. Due to excessive excavation and habitat destruction in recent years, wild resources of Chinese herbal medicines are very limited in nature. Overharvesting has gradually exhausted wild resources, and cultivated *G. uralensis* has become an alternative source^34^. However, practices and studies have indicated that the salt tolerance of cultivated *G. uralensis* is lower than that of wild plants^11^. *G. uralensis* cultivation is widely distributed in arid and semiarid desert steppes and edges and loess of hilly regions and environments^35^. The growth and development of the plant is inhibited by the inherent soil salinity associated with these areas. Enhancing the salt tolerance of *G. uralensis* in artificial cultivation systems remains a challenge that calls for urgent solutions. Considering the vital role of C and N metabolism in the plant stress response and management, this research seeks to elucidate the effect of Si on the contents of C and N metabolites, activities, and key enzymes (GS, NR, SS, and SPS) under NaCl stress and comprehensively analyze their relationship to C and N metabolism. This research also seeks to explore the role of Si in the metabolic pathways underlying salt-tolerance mechanisms to provide a scientific basis for the salt tolerance of *G. uralensis* under artificial cultivation with high growth and yield components for sustainable development.

## Materials and methods

### Plant materials and growth conditions

This study was conducted at the Experimental Field, College of Pharmacy, Ningxia Medical University, during two *G. uralensis* growing seasons in 2014 and 2015. The plant material applied was *Glycyrrhiza uralensis* Fisch, which is planted widely in the semiarid and saline regions of Northwest China. The records for the study area show that in this region, the mean annual precipitation is 200 mm, and the mean annual temperature is 8.5 °C. Moreover, the average annual sunshine hours range from 2800 to 3000 h, and the frost-free period is approximately 185 days. The physical and chemical properties of the basic soil were determined and analyzed before the experiment, and the results showed that the soil profiles could meet the requirements of the test. Soil profiles in the experimental plots consist of initial Si, total soluble salt, total nitrogen, total phosphorus, total potassium, organic matter, available nitrogen, available phosphorus and available potassium.

## Experimental design

### Plant materials and growth conditions

The experiment was randomly arranged and consisted of three salt treatments (3, 6 and 9 g NaCl/kg dry soil such as 21, 42 and 63 ds/m (NaCl)) and four Si concentration treatments (Si source using K_2_SiO_3_ as a treatment factor, with amounts of 0, 0.2, 0.4, 0.6 g SiO_2_/kg dry soil such as 0, 1.4, 2.8, and 4.2 ds/m (SiO_2_)). There were twelve treatments (3 salt treatments × 4 Si concentrations) with three replications. The salt treatments are denoted as Na_1_, Na_2_ and Na_3_, while the Si concentrations are denoted as Si_0_, Si_1_, Si_2_ and Si_3_. In the experiment, the K^+^ concentration introduced by K_2_SiO_3_ was used to maintain the consistency of the K^+^ concentration and prevent the difference in seed osmotic pressure due to the difference in K^+^ concentration, while the nutrient effect on the plant by Cl^−^ was negligible when the Cl^−^ concentration was low^36^. The soil was first put into the flowerpot after thorough mixing with NaCl, KCl and K_2_SiO_3_. The annual licorice seedlings were transplanted into flowerpots in a general trend with a consistent management practice in April 2014. Each flowerpot consisted of eight (8) strains of licorice seedlings. After transplanting, the licorice was quantitatively irrigated once (1) every seven (7) days. The pot experiment was conducted in open air to enable plants to adapt to local climatic conditions. After growing for 105 days, from mid-July to mid-October, 3 sampling frequencies (vegetative growing, flowering, maturity) were carried out. Plants were collected as samples to determine growth parameters and physiological and biochemical characteristics. From mid-July to mid-October, 3 sampling frequencies were carried out. All treatments for salt and Si were supplemented in April in the subsequent year. Si_0_ was used as a control to measure the indexes.

### Field management

Soils from the licorice planting base were collected from the farmer’s field and mixed with organic fertilizer after sieving. Plastic flowerpots 30 cm in diameter and 30 cm in height were filled with 20 kg of soil each on April 7, 2014. The 12 flowerpots were arranged on a plot and replicated three times in each of the two growing seasons. An alley of 35 cm was created to permit convenient management of the flowerpots. To create good transplant conditions for licorice, soils in the flowerpots were irrigated 4 times, 2 in the morning and 2 in the evening, at regular time intervals from April 10 to 12. Quantitatively, 2000 ml of water was used to irrigate the soil before transplanting. Eight (8) licorice seedlings that were healthy and of the same size were selected for transplantation into flowerpots on April 13.

### C metabolism-related enzyme extraction and assays

Fresh leaves tissue 0.5 g were homogenised and extracted at 4 ℃ with mortar and pestle. The extraction Tris–HCl buffer (pH 7.5) of 100 mM containing the MgCl_2_ (10 mM), EDTA-Na_2_ (1 mM), β-hydrophobic base ethanol (10 mM), 1% polyvinyl pyrrolidone and 2% ethylene glycol. The homogenate centrifuged at 15,000×*g* for 15 min^37^. The supernatant was used for the sucrose synthase and sucrose phosphate synthase assay.

Sucrose synthase was assayed in a mixed solution contained 100 mM Tris–HCl buffer (pH 7.5), 5 mM uridine diphosphate glucose (UDPG), 10 mM MgCl_2_, 5 mM fructose 6-phosphate, and 0.3 mL of the sample. The reaction mixtures were allowed to stand for 30 min at 37 ℃, 200 µl of 30% (w/v) KOH added to terminate the reactions. Placing the tubes in water bath for 10 min to destroying the remaining fructose 6-phosphate and fructose. After cooling, 0.1% (w⁄v) resorcinol of 0.25 ml and H_2_SO_4_ of 2.5 ml were added to each test tube and left for 30 min. The absorbance was measured at a wavelength of 520 nm following the procedure as modified from Verma et al.^38^ and Orathai et al*.*^39^.

Sucrose phosphate synthase was assayed in a mixed solution contained 100 mM Tris–HCl buffer (pH 7.5), MgCl_2_ (10 mM), EDTA-Na_2_ (1 mM), β-hydrophobic base ethanol (10 mM), 1% polyvinyl pyrrolidone and 2% ethylene glycol, and the 0.3 ml of sample. The reaction mixtures were incubated at 37 ℃. And 200 µl of 30% (w/v) KOH added to stop the reactions at 0 and 30 min. Putting the tubes incubated for a further 10 min at 95 ℃ to destroy any unreacted hexose phosphates. After cooling, adding mixture of anthrone reagent (in H_2_SO_4_), the absorbance was measured at wavelength 620 nm following a procedure modified from Orathai et al*.*^39^ and Charles et al*.*^40^*.*

### Determination of total sugar

The total sugar content was determined via the phenol–sulfuric acid method while using glucose as a standard^41^. The powder sample (0.0500 g) was extracted in an ultrasonic bath with 80% ethanol for 90 min at 40 °C and then diluted to a 10 ml volumetric flask, and the supernatant was the sample extract. Forty microliters of extract contained 0.5 ml of 5% phenol solution; then, 2.5 ml of concentrated sulfuric acid was added, and the absorbance of the supernatant at 490 nm was measured.

### N metabolism-related enzyme extraction and assays

GS activity were homogenized with10 mM Tris–HCl buffer (pH 7.6, containing 1 mM MgCl_2_, 1 mM EDTA and 1 mM 2-mercaptoethanol) and centrifuged at 15,000×*g* for 10 min at 4 °C. The supernatant as enzyme extract was used for GS assay. GS activity was assayed by monitoring the formation of glutamyl hydroxamate at 540 nm after reacting with 2 ml of 2.5% (w/v) FeCl_3_ and 5% (w/v) trichloroacetic acid^42^.

NR activity in the freshly harvested leaf samples (0.2 g) was determined by using the procedure of Patel et al*.*^43^ and Liu et al*.*^44^. Fresh leaves were homogenized with 5 mM phosphate buffered saline (PBS) (pH 7.5) containing 10 mM l-cysteine and 1 mM EDTA-Na_2_ and centrifuged at 12,000×*g* for 10 min at 4 ℃. The supernatant was used for NR assay. The control treatments without NADH. The reaction was halted by adding 1% (w/v) sulfanilamide and 0.02% (w/v) of N-(1-naphthyl) ethylene diamine dihydrochloride. After develop a pink color to quantify photometrically at 540 nm.

### Determination of total nitrogen

The total nitrogen content was analyzed from 2 g dried leaves which ground with a mortar and a pestle. The sample was digested in a Kjeldahl method^45^ with 20 ml of concentrated H_2_SO_4_ and 0.2 g KMNO_4_ (catalyst).

### Determination of Si contents

The Si content was determined using the dry ashing and molybdenum blue colorimetric method^46^. Approximately 100 mg of finely ground plant sample was placed in a nickel crucible, and NaOH was added to each crucible at a high burning temperature for 15 min. The high temperature, however, oxidized all the organic matter present and solubilized the Si in the muffle furnace when kept for approximately 2 h. Discoloration development was achieved by adding 35 ml of 20% acetic acid to 10 ml of ammonium molybdate solution, 5 ml of 20% tartaric acid, 1 ml of reducing solution and 20% acetic acid in a volume of 50 ml. After mixing, the optical density was read at 630 nm with a spectrophotometer.

### Statistics analysis

All experimental data were analyzed by ANOVA using SPSS 17.0 software (SPSS Inc., USA), and significant differences were tested using the least significant differences (LSD) test at P < 0.05. Mean values and standard errors (SE) are presented. The figures were prepared using Microsoft Excel 2016.

## Results

### Effect of Si addition on the SS and SPS activity of *G. uralensis* under NaCl stress

**Figure 1:**
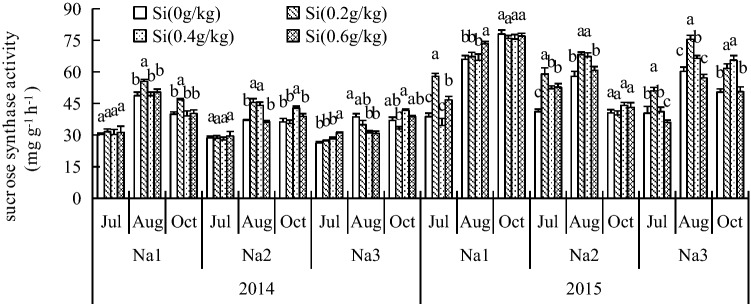
Effect of silicon addition on the SS activity of *G. uralensis* under NaCl stress. The different letters within the different treatments indicate the significant difference at P ≤ 0.05 (Si source using K_2_SiO_3_ as a treatment factor, with amounts of 0, 0.2, 0.4, 0.6 g SiO_2_/kg dry soil). The salt treatments are denoted as Na_1_, Na_2_ and Na_3_, while the Si concentrations are denoted as Si_0_, Si_1_, Si_2_ and Si_3_).

**Figure 2:**
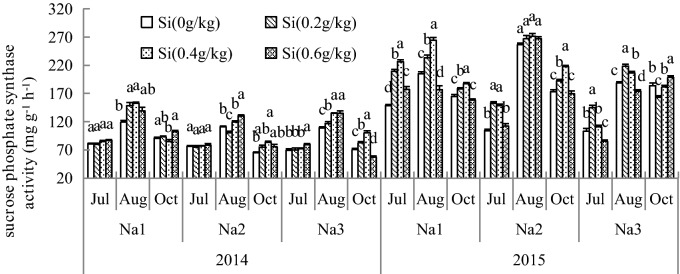
Effect of silicon addition on the SPS activity of *G. uralensis* under NaCl stress.The different letters within the different treatments indicate the significant difference at P ≤ 0.05 (Si source using K_2_SiO_3_ as a treatment factor, with amounts of 0, 0.2, 0.4, 0.6 g SiO_2_/kg dry soil). The salt treatments are denoted as Na_1_, Na_2_ and Na_3_, while the Si concentrations are denoted as Si_0_, Si_1_, Si_2_ and Si_3_).

In this experiment, we found significant increases in SS and SPS activities between + Si treatments. The significant effect was concentration-dependent (Figs. 1 and 2). The activities of SS and SPS in *G. uralensis* leaves were significantly decreased after NaCl was added compared to those in plants grown with Na_1_ (CK). However, SS activity was significantly intensified with Si_2_ over the experimental period compared to plants grown without Si (Si_0_) in both years. SPS activity was significantly intensified also with Si_2_ in both years, mostly after treatment, compared to plants grown without Si.

### Effect of Si addition on the carbon metabolite total sugar of *G. uralensis* under NaCl stress

**Figure 3:**
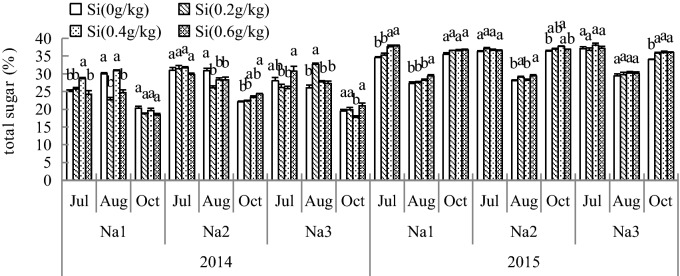
Effect of silicon addition on the carbon metabolites total sugar of *G. uralensis* under NaCl stress.The different letters within the different treatments indicate the significant difference at P ≤ 0.05 (Si source using K_2_SiO_3_ as a treatment factor, with amounts of 0, 0.2, 0.4, 0.6 g SiO_2_/kg dry soil). The salt treatments are denoted as Na_1_, Na_2_ and Na_3_, while the Si concentrations are denoted as Si_0_, Si_1_, Si_2_ and Si_3_).

From the total sugar data over 2 years, it was observed that the total sugars of roots were increased by Si under salt stress, as shown in Fig. 3. The addition of Si could affect the content of total sugar; compared to plants grown with Na_1_ (CK), the content of total sugar was significantly intensified with Si_2_ and Si_3_. After adding Si, the total sugar increased to 14.38% in the Si_2_ treatment (2.8 ds/m). With increasing Si concentration, the total sugar content gradually increased to 25.70% under salt stress.

### Effect of Si addition on the GS and NR activity of *G. uralensis* under NaCl stress

**Figure 4:**
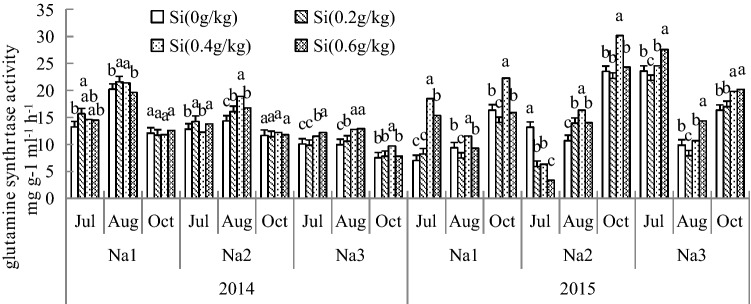
Effect of silicon addition on the GS activity of *G. uralensis* under NaCl stress.The different letters within the different treatments indicate the significant difference at P ≤ 0.05 (Si source using K_2_SiO_3_ as a treatment factor, with amounts of 0, 0.2, 0.4, 0.6 g SiO_2_/kg dry soil). The salt treatments are denoted as Na_1_, Na_2_ and Na_3_, while the Si concentrations are denoted as Si_0_, Si_1_, Si_2_ and Si_3_).

**Figure 5:**
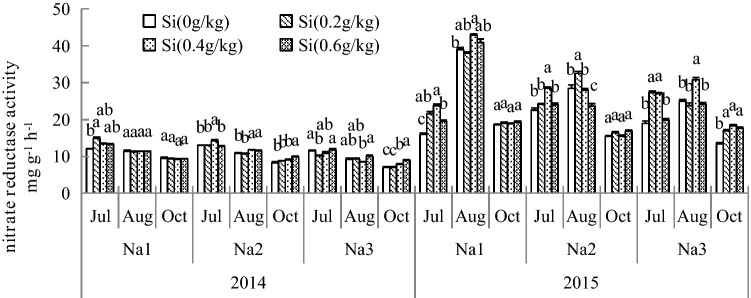
Effect of silicon addition on the NR activity of *G. uralensis* under NaCl stress.The different letters within the different treatments indicate the significant difference at P ≤ 0.05 (Si source using K_2_SiO_3_ as a treatment factor, with amounts of 0, 0.2, 0.4, 0.6 g SiO_2_/kg dry soil). The salt treatments are denoted as Na_1_, Na_2_ and Na_3_, while the Si concentrations are denoted as Si_0_, Si_1_, Si_2_ and Si_3_).

The results show that Si can increase GS and NR enzyme activity in 2 years, as shown in Figs. 4 and 5. The NR and GS activities were significantly increased in leaves after adding Si under salt stress. The maximum activity was recorded at 2.8 ds/m Si treatment, and the GS and NR activities were increased by 163.34% and 42.02%, respectively, compared with those of the control. The increase in added Si under salt stress was more severe for GS activity than NR activity. Under salt stress, the activities of GS and NR were significantly increased in leaves after adding Si in both years. The activities of these enzymes showed an upward trend with increasing duration of salt treatment and Si content.

### Effect of Si addition on the nitrogen metabolite total nitrogen of *G. uralensis* under NaCl stress

**Figure 6:**
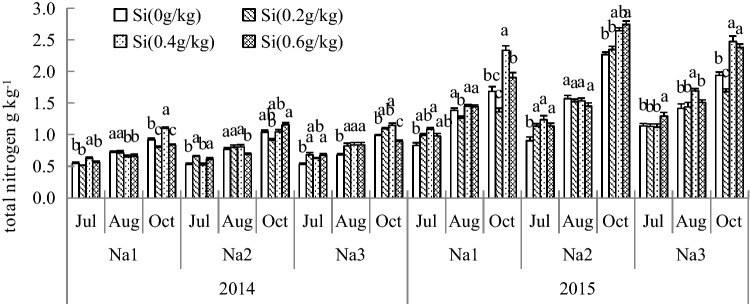
Effect of silicon addition on the nitrogen metabolites total nitrogen of *G. uralensis* under NaCl stress. The different letters within the different treatments indicate the significant difference at P ≤ 0.05 (Si source using K_2_SiO_3_ as a treatment factor, with amounts of 0, 0.2, 0.4, 0.6 g SiO_2_/kg dry soil). The salt treatments are denoted as Na_1_, Na_2_ and Na_3_, while the Si concentrations are denoted as Si_0_, Si_1_, Si_2_ and Si_3_).

As shown in Fig. 6, Si significantly increased the content of total nitrogen in roots under salt stress. And the content of total nitrogen showed an increasing trend, which increased gradually with stress time. The content of total nitrogen was significantly increased in Si_2_ or Si_3_. Adding Si under salt stress resulted in maximum increases recorded in both years about 27.18% to 38.74%, compared with the control.

### Effect of Si addition on root dry weight of *G. uralensis* under NaCl stress

**Figure 7:**
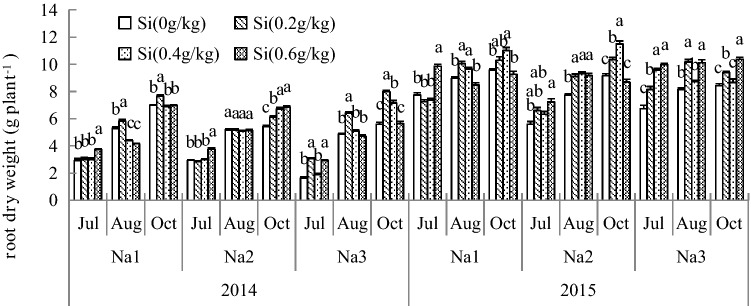
Effect of silicon addition on root dry weight of *G. uralensis* under NaCl stress.The different letters within the different treatments indicate the significant difference at P ≤ 0.05 (Si source using K_2_SiO_3_ as a treatment factor, with amounts of 0, 0.2, 0.4, 0.6 g SiO_2_/kg dry soil). The salt treatments are denoted as Na_1_, Na_2_ and Na_3_, while the Si concentrations are denoted as Si_0_, Si_1_, Si_2_ and Si_3_).

As shown in Fig. 7, the root dry weight per plant of *G. uralensis* in 2015 was significantly larger than that in 2014, and the root dry weight tended to increase gradually with the passage of time. In the growth period, the application of Si significantly increased the dry weight of roots under salt stress conditions. Adding Si under salt stress resulted in maximum increases recorded of 85.39%, compared with the control in both years.

### Si content of *G. uralensis* under NaCl stress in different parts

**Figure 8:**
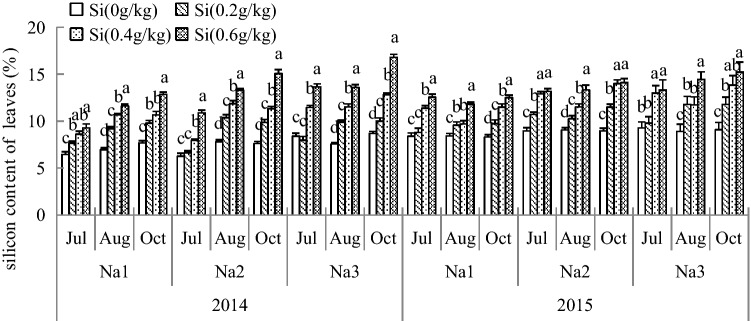
The silicon content of *G. uralensis* under NaCl stress in leaves. The different letters within the different treatments indicate the significant difference at P ≤ 0.05 (Si source using K_2_SiO_3_ as a treatment factor, with amounts of 0, 0.2, 0.4, 0.6 g SiO_2_/kg dry soil). The salt treatments are denoted as Na_1_, Na_2_ and Na_3_, while the Si concentrations are denoted as Si_0_, Si_1_, Si_2_ and Si_3_).

**Figure 9:**
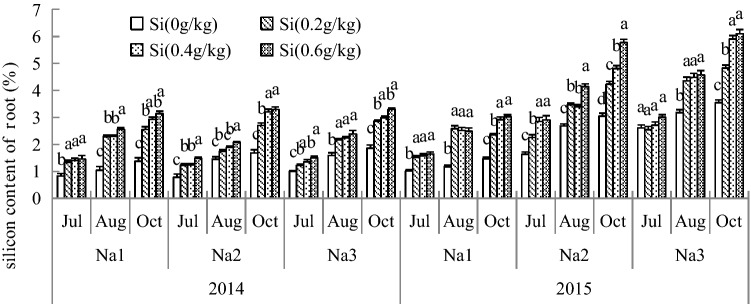
The silicon content of *G. uralensis* under NaCl stress on root. The different letters within the different treatments indicate the significant difference at P ≤  0.05 (Si source using K_2_SiO_3_ as a treatment factor, with amounts of 0, 0.2, 0.4, 0.6 g SiO_2_/kg dry soil). The salt treatments are denoted as Na_1_, Na_2_ and Na_3_, while the Si concentrations are denoted as Si_0_, Si_1_, Si_2_ and Si_3_).

**Figure 10:**
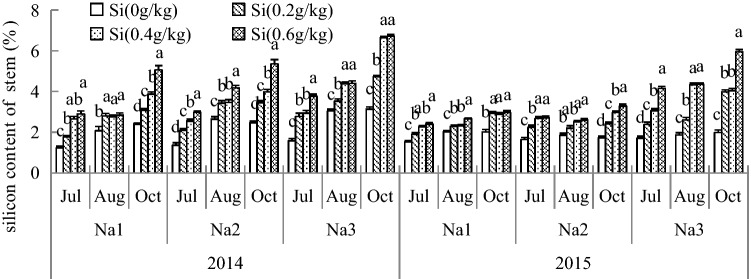
The silicon content of *G. uralensis* under NaCl stress in stem. The different letters within the different treatments indicate the significant difference at P ≤  0.05 (Si source using K_2_SiO_3_ as a treatment factor, with amounts of 0, 0.2, 0.4, 0.6 g SiO_2_/kg dry soil). The salt treatments are denoted as Na_1_, Na_2_ and Na_3_, while the Si concentrations are denoted as Si_0_, Si_1_, Si_2_ and Si_3_).

Plants absorb and utilize Si differently in different parts. As shown in Figs. 8, 9 and 10, under the different treatments, the contents of Si in the roots, stems and leaves were different, but the content of Si reached a maximum with the Si_3_ treatment and was significantly larger than that with the Si_0_ treatment. Additionally, the accumulation of Si in the root was smaller, and the content of Si was highest in the leaves.

### Correlation analysis of Si and related indexes of *G. uralensis* under NaCl stress

As Table 1 indicates, we performed a correlative analysis between *G. uralensis* dry weight of the root and the enzyme activity in C and N metabolism as well as C and N metabolites; the result showed a strong innate connection between the *G. uralensis* dry weight of the root and each index. The largest influences are conferred by root Si content and leaf total nitrogen of *G. uralensis.* According to the correlation coefficient, the different metabolites showed apparent positive correlations with related enzyme activity.

**Table 1:** The correlation analysis on the silicon and related indexes of *G. uralensis* under NaCl stress.

	SS activity	SPS activity	GS activity	Total sugar	Total nitrogen	NR activity	dry weight	Leaves silicon	Stem silicon	Root silicon
SS activity		0.734**	− 0.070	0.286*	0.554**	0.663**	0.708**	0.173	− 0.099	0.421**
SPS activity			0.147	0.406**	0.621**	0.717**	0.701**	0.190	− 0.210	0.467**
GS activity				0.424**	0.406**	− 0.073	0.263*	0.180	− 0.030	0.306**
Total sugar					0.479**	0.411**	0.399**	0.148	− 0.336**	0.225
Total nitrogen						0.374**	0.796**	0.404**	0.092	0.791**
NR activity							0.534**	0.078	− 0.323**	0.205
Dry weight								0.388**	0.106	0.658**
Leaves silicon									0.709**	0.675**
Stem silicon										0.460**
Root silicon										

**Means significant differences by Duncan’s multiple range tests (P < 0.01); *means significant differences by Duncan’s multiple range tests (P < 0.05).

### Path analysis of yield and Si content and carbon and nitrogen metabolites of *Glycyrrhiza uralensis* Fisch

The dry matter content, Si content and carbon and nitrogen metabolites in the roots of *G. uralensis* were analyzed by SPSS path analysis. The independent variables were set as follows: Y_1_: dry matter mass per plant (g), X_1_: Si content in root, X_2_: total sugar and X_3_: total nitrogen. The stepwise regression equation and partial correlation coefficient are as follows: Y_1_ = 0.328X_1_ + 0.029X_2_ + 2.742X_3_ + 1.062, F = 39.434. The direct and indirect effects of the above factors on yield were obtained by path analysis with dry matter per plant of licorice, as shown in Tables 2, 3 and 4:

**Table 2:** The direct and indirect effect from silicon content, carbon and nitrogen metabolites to root dry matter of *G. uralensis.*

Independent variable	Correlation coefficient r_i0_	Direct action p_0i_	Indirect action			
			Total	Through		
				X_1_	X_2_	X_3_
X_1_	0.679**	0.181	0.496		0.024	0.466
X_2_	0.433**	0.066	0.367	0.065		0.325
X_3_	0.776**	0.607**	0.169	0.139	0.034	

**Means significant differences by Duncan’s multiple range tests (P < 0.01); *means significant differences by Duncan’s multiple range tests (P < 0.05). Y_1_: dry matter mass per plant (g), X_1_: Si content in root, X_2_: total sugar and X_3_: total nitrogen.

**Table 3:** The coefficient of determination from silicon content, carbon and nitrogen metabolites to root dry matter of *G. uralensis.*

	X_1_	X_2_	X_3_
X_1_	0.033		
X_2_	0.009	0.004	
X_3_	0.169	0.042	0.368

X_1_: Si content in root, X_2_: total sugar and X_3_: total nitrogen.

**Table 4:** The total contribution from silicon content, carbon and nitrogen metabolites to root dry matter of *G. uralensis.*

	X_1_	X_2_	X_3_
Y_1_	0.1228	0.0285	0.4710

Y_1_: dry matter mass per plant (g), X_1_: Si content in root, X_2_: total sugar and X_3_: total nitrogen.

The determinant coefficients were arranged by calculating the absolute values, and the effects on the dry matter content of the roots of *G. uralensis* were as follows: X_3_ > X_1_X_3_ > X_2_X_3_ > X_1_ > X_1_X_2_ > X_2_.

The R_2_ total contribution of each variable was analyzed, and the reliability of the regression equation was estimated. The calculation results are shown in Table 4. It was found that the total contribution of the independent variable total nitrogen to R_2_ was 0.4710, ranking first in the total contribution of the independent variable to R_2_, followed by Si content and total sugar. Therefore, aggregate analysis of the yield of *G. uralensis* roots revealed the following:

With the increase in Si content X_1_, root yield Y increased directly (0.181), root yield X_1_ increased, total sugar (0.024) and total nitrogen (0.466) increased, root yield Y increased to a certain extent (0.496), and the final comprehensive yield increased significantly (0.679).

With the increase of total sugar content X_2_, root yield Y increased directly (0.066), indirectly affecting the increase of Si content X_1_ and total nitrogen content X_2_; promotion of root yield Y increased (0.065 and 0.325) total sugar (-0.013) decreased, ultimately promoting root yield to a certain extent (0.367); and ultimately overall yield increased significantly (0.433).

With the increase in total nitrogen content X_3_, root yield Y increased significantly (0.607) and X_3_ increased, while affecting root Si content (0.139); total sugar content increased (0.034), ultimately promoting root yield to a certain extent (0.169); and ultimately total yield increased significantly (0.776).

It was found that total nitrogen played a large role in the accumulation of *G. uralensis.* The total nitrogen content also depends on the quality of other components. Under NaCl stress, adding Si treatment can adapt to environmental changes by adjusting its physiological process, promoting material accumulation and increasing the yield of *G. uralensis* per plant.

### Multiple linear regression of Si content and root dry matter mass of *G. uralensis* under NaCl stress

**Figure 11:**
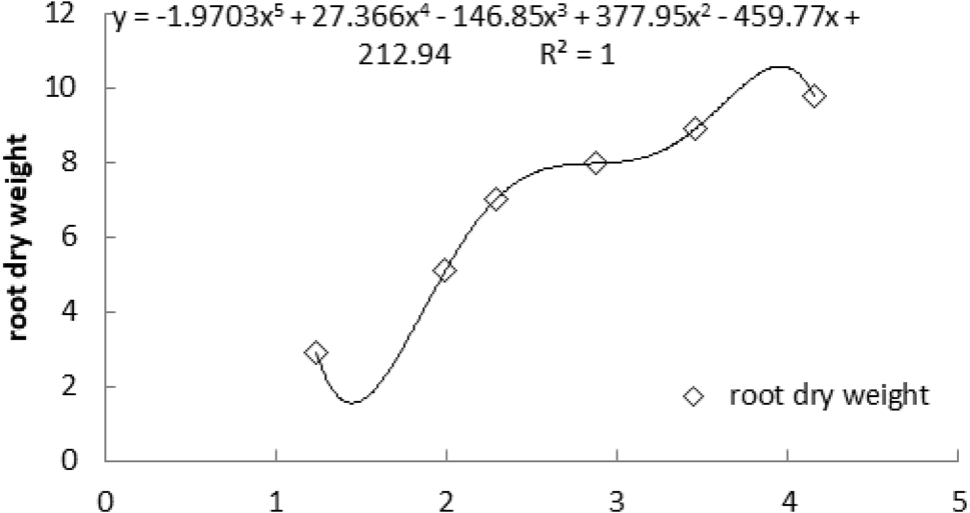
The multiple linear regression about silicon content and root dry matter mass of *G. uralensis* under NaCl stress.

As shown in Fig. 11, the abscissa coordinates represent the content of Si at different times. The determination times were July 20, August 20, October 20, 2014, and July 20, August 30, and October 10, 2015, and the Si contents (%) were 1.23, 1.98, 2.29, 2.64, 3.56 and 4.29, respectively. From August 2014 to July 2015, the accumulation of Si in licorice roots remained basically unchanged and increased rapidly starting in August 2015, which was basically synchronized with the accumulation of dry weight per plant in the figure. Multiple linear regression analysis of Si content and dry weight per licorice root plant was carried out, and the regression equation was obtained. When the concentration of Si was 4.00946%, the dry weight per plant of licorice root reached the maximum, which was 10.92946 g.

## Discussion

Under salt stress, the enzymes of C and N metabolism were significantly altered, and these harmful effects decreased plant growth. C and N metabolism are basic metabolic processes in the growth and development of plants that ensure the normal growth and development of crops and the yield and quality^47^. Shao reported that salt stress-induced growth inhibition in cucumber seedlings may involve disruption of nitrogen absorption and decreased activities of enzymes associated with nitrogen assimilation^48^. Irani et al*.* showed that salt stress induces metabolic disorders of nitrogenous compounds in *Arabidopsis thaliana*^49^. In addition, it has been reported that the activities of NR and GS decrease under salt stress, influencing the transformation and utilization of nitrogen in plants. In this study suggested that Si supplied may be beneficial to offset this harmful effect of NaCl stress on *G. uralensis* growth, as reported previously in mung bean^50^, barley^51^, rice^52^, cherry tomatoes^53^, and *Cucumis sativus* L.^54^. When salt stress were 21 and 42 ds/m, the activity of the carbon metabolizing enzymes SPS and SS in the leaves of *G. uralensis* significantly increased in Si_1_ and Si_2_. The activities of SPS and SS enzymes were stronger in Si_2_ and Si_3_ under high salt stress. Thus, it can be seen that Si may act as a signaling factor redirecting the primary metabolism of plants by increased the SS and SPS enzyme activity, allowing synthesis and accumulation of carbon metabolites. Although adding Si had no remarkable influence on the products of metabolism, Si enhanced the content of total sugar under NaCl stress when its concentration was 42 ds/m. it may enhanced tolerance to osmotic stress under salt deficiency. Therefore, adding Si regulated processes including C and N metabolism of *G. uralensis* under stress, which would significantly increase enzyme activity, alleviate stress damage, and increase the synthesis and accumulation of metabolite production. The synthesis and accumulation of metabolite production reflecting the tolerance of *G. uralensis* to osmotic stress. Our investigation reveals an important role of Si in C and N metabolism under salt stress. Therefore, by adding a suitable concentration of Si and allowing pertinent regulation of enzymatic reactions and C, N metabolism promotes the coordination of *G. uralensis* development and all metabolic activities and increases yield and quality.

SS and SPS play important roles as developmental regulators of C metabolic processes in plants. SS and SPS are important enzymes in C metabolism capacity, and their activity determines primary metabolite enrichment. Salt stress affected the activities of SS and SPS in *Lupinus albus* L.^55^. In the present study, increases in SS and SPS activity were observed in Si under salt-stressed *G. uralensis*, with a resulting increase in sucrose content, indicating that Si can buffer fluctuations in carbohydrate contents in response to osmotic stress caused by salt stress. These results indicated that Si further mitigates salt stress by inducing SS and SPS activities in salt-stressed *G. uralensis*; correspondingly, these enzymes can affect carbohydrate levels. N assimilation is also an important physiological metabolic process, and plants do not absorb inorganic N from soil but change it into organic N through a series of physiological and biochemical reactions, allowing it to be absorbed and utilized by plants. Salt stress not only alters the N absorption of plants but affects the activities of key enzymes in the N assimilation pathway, which causes an imbalance in N metabolism^48^. Previous studies showed that NR activity was reduced in salt-stressed Ethiopian mustard^8^. In the present study, salt stress significantly reduced NR activity in *G. uralensis*, while Si application remarkably enhanced NR activity in salt-stressed *G. uralensis*. These results indicate that Si can mitigate salinity-induced effects, resulting in an increase in NR activity. GS is vital in plants and is also the major enzyme for ammonium assimilation, and the final form of endogenous inorganic N is ammonium^56^. Our results showed that salt stress decreased GS activities in *G. uralensis*, which is similar to the results in cashews^57^. These results indicated that GS activity is strongly limited in plants exposed to saline conditions. However, Si application significantly increased GS activity in salt-stressed *G. uralensis*, indicating that Si could improve the activities of enzymes to help maintain the N metabolism balance in salt-stressed *G. uralensis*. Thus, adding Si at an optimal concentration in favor of C and N metabolism can be carried out normally under salt stress and establish a foundation to obtain excellent characteristics of *G. uralensis*.

Si is beneficial mostly for higher plants, especially under stressful environments, and the addition of Si to the growth medium resulted in significantly higher Si accumulation in plant tissues observed a significant decrease in Si content of tomato roots under salt stress, indicating that salt stress significantly affected the accumulation of Si^22,58,59^. The experiment was conducted to determine Si accumulation in different parts of *G. uralensis* primarily to determine the distribution of Si in plants. In the present study, the concentrations of Si in plants were higher than those without Si treatment under the same NaCl stress. The study also found that the content of Si in the leaves was far greater than that in the roots. These results indicated that leaves and roots differ in their abilities to accumulate Si. Also, the beneficial effects provided by Si are closely correlated with the Si accumulation level in *G. uralensis* plants. The content of Si was higher in shoot, probably due to Si-enhanced biomass production. Therefore, we conclude that *G. uralensis* is taken up Si in below-ground parts, and most part is translocated to the shoot. The beneficial effects provided by Si are closely correlated with the Si accumulation level in *G. uralensis* plants, which may be an adaptive mechanism for *G. uralensis* to moderate salt stress by taking up and transporting more Si, further promoting plant growth under salt stress.

Si can positively influence plant growth and yield, as shown in numerous studies^60,61^. The results of this study indicate that the quality and yield of *G. uralensis* were significantly increased. The dry weight of *G. uralensis* roots was increased by 12.8–64.07% by adding Si under different concentrations of NaCl stress. Si can diminish the inhibitory effect of salt stress on plant growth. Additionally, correlative analysis between *G. uralensis* dry weight of roots and the enzyme activity in C and N metabolism as well as C and N metabolites shows that the root Si content has the biggest influence on the dry weight of roots. These results indicated that Si can increase enzyme activities and provoke the accumulation of metabolites in *G. uralensis* plants. All these results indicate that Si is indispensable and important for *G. uralensis* under salt stress, and is taken up by the roots, transported via the xylem in the transpiration stream and distributed within plant tissues. In leaf sheaths and leaf blades silicic acid polymerizes into amorphous silica, which is deposited into the cell wall, cell lumen, intracellular spaces and trichomes increasing tissue strength, which might improve the structure of plant leaves to alleviate damage to the photosynthetic system under NaCl stress in *G. uralensis*. Further research on the possible mechanisms of Si uptake and transport in *G. uralensis* is extremely important to utilize Si-induced beneficial effects, which need further research in the future.

## Conclusion

In conclusion, Si regulates C and N metabolism and alters physiological activities, particularly in plants subjected to NaCl stress conditions, which could alleviate adverse effects induced by salt stress on *G. uralensis*. Specifically, Si significantly increased the total sugar and total N contents in *G. uralensis* due to balanced regulation of related enzymes, and the increase in total sugar and total N also played a protective role in osmoregulation under salt stress. Si could alleviate the adverse effects of salt stress and then improve plant growth. Therefore, we studied the relationship between Si and C and N metabolism under different concentrations of NaCl, pertinently regulating key enzyme activity through external conditions, to obtain optima for the C–N metabolism of *G. uralensis*. Additionally, this test provides a theoretical basis for the actual production of *G. uralensis* to improve yield and quality, which has very important practical value to improve the absorbability of *G. uralensis* in saline. Future research should further explore the possible mechanisms of Si uptake and transport in *G. uralensis* to exploit Si-induced beneficial effects. This will allow us to better understand the interactions between Si application and plant responses. This information will be used in fertilization practices to enhance stress tolerance in crop systems.

## Data Availability

The datasets used and/or analyzed during the current study are available from the corresponding author on reasonable request.

## Abbreviations

Si: Silicon
SPS: Sucrose phosphate synthetase
SS: Sucrose synthetase
NR: Nitrate reductase
GS: Glutamine synthetase
